# Advances in the Diagnosis of Reproductive Disorders in Male Camelids

**DOI:** 10.3390/ani15192931

**Published:** 2025-10-09

**Authors:** Abdelmalek Sghiri, Muhammad Salman Waqas, Michela Ciccarelli, Abelhaq Anouassi, Ahmed Tibary

**Affiliations:** 1 Medicine, Surgery and Reproduction Department, Institut Agronomique et Vétérinaire Hassan II, Rabat-Institut, Rabat 6202, Morocco; asghiri6119@gmail.com; 2 Department of Veterinary Clinical Sciences, College of Veterinary Medicine, Washington State University, Pullman, WA 99164, USA; michela.ciccarelli@wsu.edu (M.C.); tibary@wsu.edu (A.T.); 3 Advanced Scientific Group, Sweihan, Abu Dhabi P.O. Box 45803, United Arab Emirates; anouassi@yahoo.com

**Keywords:** phimosis, libido, azoospermia, teratozoospermia, subfertility, penis, prepuce

## Abstract

Male fertility is important to ensure herd health and productivity. The camelid male breeding soundness examination (BSE) is strongly recommended because natural mating remains the primary breeding method due to the challenges in semen cryopreservation and artificial insemination. Guidelines for the BSE have been proposed but not adopted in practice. The investigation of male reproductive failure includes history, general health examination, examination of the genitalia, semen evaluation, and testing for contagious diseases. Difficulties in ejaculate collection and semen viscosity are challenges in camelid male fertility investigation. This review summarizes the outcomes of BSE in our practice on South American camelids (SACs) and camels. The results and discussion are presented under four main categories: congenital defects, *impotentia coeundi, impotentia generandi*, and male reproductive emergencies. There is a difference between camels and SACs in the incidence of various disorders. Congenital defects are common in SACs in particular cryptorchidism, testicular hypoplasia and rete testis cysts. Orchitis is more common in camels, particularly in areas where brucellosis is prevalent. Testicular degeneration occurs in all camelids and has been associated with heat stress, aging, systemic diseases and overuse of anabolic steroids. Precise diagnosis of fertility impairment may require disease testing, testicular biopsy, cytogenetics and endocrine evaluation. A significant proportion of males are referred because of reproductive emergencies, due to poor management, which results in loss of genetic potential. Implementation of regular BSE is possible in SACs but can be difficult in dromedaries because of the large variation in breed characteristics and management systems.

## Simple Summary

Male fertility and breeding soundness examination are important for all domestic animals. The article emphasizes fertility evaluation and the diagnostic approach to male fertility in camelids. Camelid male breeding soundness examination is strongly recommended. This review summarizes the outcomes of breeding soundness examinations under four main categories of congenital reproductive defects, impotentia coeundi, impotentia generandi and male reproductive emergencies. The diagnostic procedure to evaluate male fertility includes history, examination of the genitalia, ultrasonography of the reproductive tract, semen collection and evaluation, and testing for contagious diseases. Semen viscosity hinders accurate sperm motility and concentration evaluation, requiring enzymatic treatment of the ejaculate. Common reproductive disorders found in our clinical cases include rete testis cysts, testicular hypoplasia, testicular degeneration, and hydrocele. Hormonal and cytogenetic testing can be useful and are indicated in some cases. Besides veterinary medical and biological aspects, breeding male management through adequate health, nutrition, housing, and sexual use for mating or semen collection is crucial for ensuring male fertility and preventing reproductive disorders.

## 1. Introduction

Numerous studies in cattle, sheep, and goats have shown the importance of male fertility for herd health and reproductive efficiency. In addition, new male introduction into a herd poses a significant biosecurity risk unless they are tested for contagious diseases. Guidelines have been established for bovine and small ruminants breeding soundness examination and have led to a significant improvement in reproductive performance [1,2].

The interest in camelid breeding has increased in recent years with the introduction of South American Camelids (SACs) to North America, Australia, New Zealand, and Europe, alongside the development of dromedary racing and dairy industries. These activities make the male camelid an essential part of the herd’s genetic improvement. Elite sires are in high demand for breeding, making their reproductive capacity extremely important. Male fertility is crucial as natural mating remains the predominant breeding method because of the challenges encountered in the implementation of semen cryopreservation and artificial insemination in camelids [3,4,5]. While good pregnancy rates have been obtained following artificial insemination with fresh diluted semen results, the results are low and variable with the use of cooled semen (dromedary [5,6], SAC [4]).

Three main camelid breeding systems exist: free-mating system, small group mating, and in-hand breeding. The free mating system, usually referred to as the traditional or extensive breeding system, is the primary method of breeding in large herds of camels in desert conditions [7]. Field surveys of the traditional camel rearing systems report a male-to-female ratio ranging from 1:25 to 1:100 [7,8]. A variation of the free mating system exists in llama and alpaca herds, where a group of males is introduced to a herd of several females. The males are changed every 2 to 3 weeks. The small group mating system consists of one male joined to a small group of females. This system is used in dairy camels, alpacas, and llamas. The in-hand breeding system is mainly used in high-end breeding facilities where the males are in high demand (SAC in North America, Europe, or Australia; racing and show camels in the Middle East). In dromedary herds, calving rates in extensive systems range from 30 to 60% [7,9], whereas in well-managed herds, this rate can reach 80 to 90% [8]. In alpacas, the seasonal pregnancy rate in the traditional Peruvian system (breeding season of 60 to 90 days) is between 40 and 60% [10]. In one study in New Zealand, the pregnancy rate of alpacas mated in spring and autumn was 22% and 61%, respectively [11]. Under good nutritional management and veterinary care, the pregnancy rate in North American alpaca and llama herds varies from 65 to 95% (Tibary A, personal observation). Herd pregnancy rates differ significantly because of differences in breeding systems, variation in the length of the breeding season, nutritional status and disease prevalence. Therefore, it is difficult to determine the role a male plays in the overall reproductive performance of the herd.

Although some guidelines have been proposed for camelids, regular male breeding soundness examinations are not performed in most breeding operations. Males are often presented to the veterinarian when the owner suspects a fertility problem or notices a change in the behavior or the external genitalia.

There are limited reports on reproductive pathology in male Camelidae. The authors have emphasized the importance of conducting a breeding soundness examination both as a routine and for infertile males over the last 35 years. This has led to the accumulation of clinical information on the most common reproductive disorders, which have been available only through postmortem observations (dromedary [12,13] and alpaca [14]. The present paper aims to discuss the results of male camelid breeding soundness examinations in veterinary clinical practice and describe advances in the diagnosis of reproductive disorders in camelids.

## 2. Breeding Soundness Examination Results

A male camelid breeding soundness examination (BSE) protocol was proposed in 1997 [15] and updated in 2024 [16]. Similar approaches have also been published for dromedaries [17,18]. The male BSE is not limited to the examination of the genitalia but also includes general health, testing for contagious diseases, and examination for any congenital disorders that may affect reproductive ability or be hereditary [15,19].

Heritable congenital defects remain poorly studied in the camelids. SACs are notorious for the high incidence of congenital defects that may be hereditary [20,21]. Similar disorders have been described in camels. However, their true incidence and heritability are still not known.

**Figure 1:**
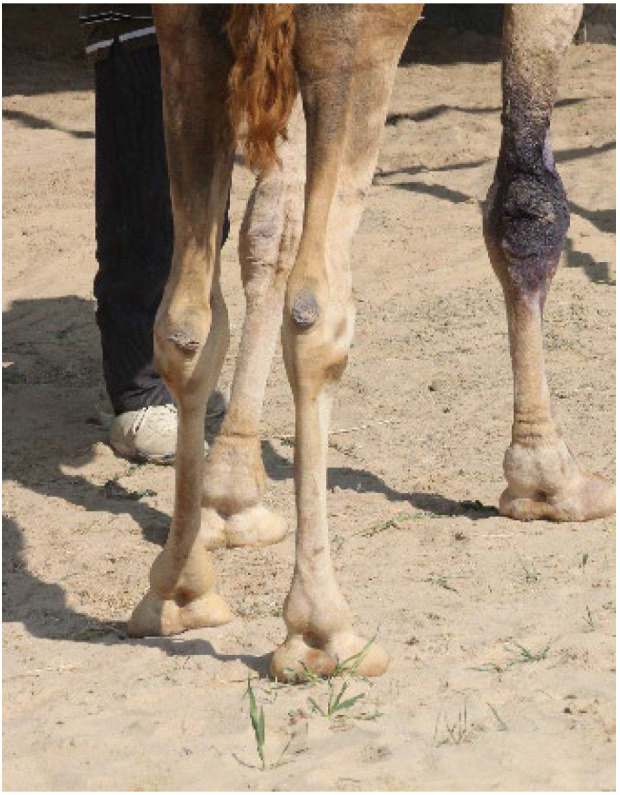
Osteoarticular problems in a retired racing male.

Reproductive success depends on physical soundness as well as the normal function of the reproductive system. Males should be in perfect body condition and health before the breeding season, particularly in camels. During the rutting season, camels undergo stress-induced metabolic and endocrine adaptations, leading to reduced food intake and a substantial decrease in body weight [22]. Given the peculiar copulatory behavior, the soundness of the musculoskeletal system is essential for mating. This is especially important in aged males or retired racing dromedaries, which are often predisposed to degenerative joint disease and lameness (Figure 1).

**Figure 2:**
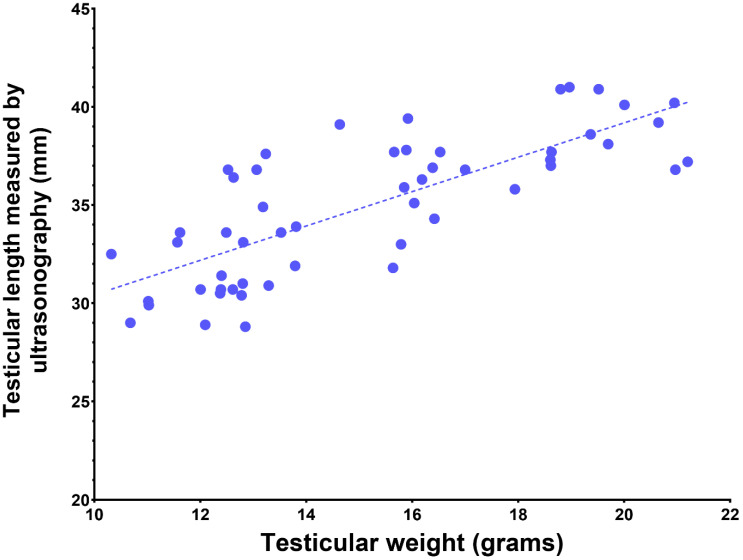
Correlation between testicular length and testicular weight in alpacas (n = 50, r = 0.8).

The examination of the reproductive system begins with the evaluation of the external genitalia. The scrotum is examined for its conformation and the presence of any lesions. The testes are evaluated for presence, symmetry, and size. In SACs, the length and width of each testicle are predictive of the volume of the testis and are well correlated to testicular weight [23,24] (Figure 2).

Age-specific minimum testicular length and width have been established for llamas and alpacas (see review [25]. In camels, scrotal circumference is positively correlated with testicular volume [26] and epididymal sperm reserves [27]. However, due to the position of the testes and the tight scrotum, particularly in young animals, this measurement is less reliable for estimation of the testicular volume compared to caliper or ultrasonographic measurement of the length and width of each testicle. Camels should be examined during the breeding season as testicular size and function undergo significant seasonal changes. Recommendations for minimum testicular size for different age groups are difficult because of significant variations reported in the literature [16,18]. Testicular size variability is likely due to differences among dromedary breeds as well as types of rearing conditions. To the authors’ knowledge, there are no studies on testicular size in the Bactrian camel.

**Figure 3:**
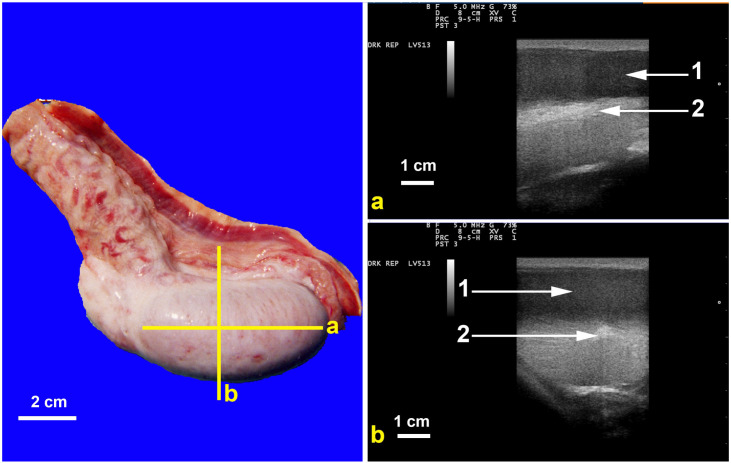
Ultrasonography of dromedary’s normal testis. In Left image (**a**) demonstrates scanning longitudinal axis while (**b**) demonstrates transducer placement for cross section of testis. In images a and b on the right, 1 normal testicular parenchyma while 2 is hyperechoic mediastinum testis.

Testicular ultrasonography should be part of the breeding soundness examination of male camelids, as many testicular and epididymal disorders cannot be detected by palpation. Testicular echotexture is similar to that of ruminants (Figure 3).

Software algorithms have been developed for ultrasound image analysis for more precise evaluation. A study on dromedary camels found a good correlation between the results of ultrasound image analysis and semen quality [28].

**Figure 4:**
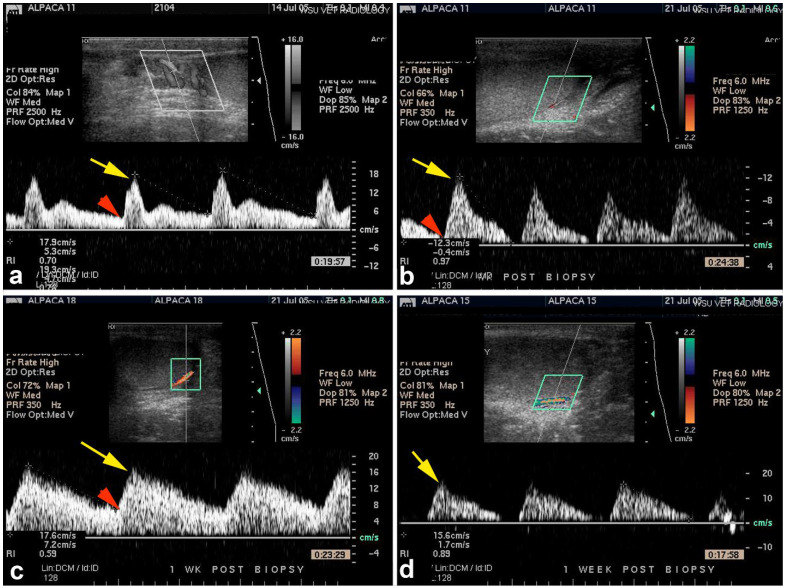
Power (**a**) and color (**b–d**) Doppler ultrasonography for the evaluation of blood flow to an alpaca testis following testicular biopsy. Each image represents a different alpaca. Velocity wave forms from four different alpacas showing variable waveform. Yellow arrows indicate peak systolic velocity and red arrow indicate end diastolic velocity.

Testicular blood flow determination using power and color Doppler ultrasonography has been described in SACs [29] (Figure 4) and dromedaries [30,31] and may provide additional information if the technique is standardized.

However, a study on a small number of alpacas did not find any correlation between testicular ultrasonography, blood flow, and pregnancy rates [32]. This study also did not find any difference in testicular volume between different age groups of males (12–14 months, 24 months, and 36 months or more).

Examination of the prepuce is relatively easy in camels but requires restraint in SACs. The examination of the penis can only be performed during copulation. For a complete examination, the penis should be exteriorized under general anesthesia or pudendal nerve block [33].

Anatomy and ultrasonography of the internal genitalia have been described in detail [16]. However, examination of these structures is only indicated in specific cases [15]. Determination of the distance from the anal sphincter to the prostate by ultrasonography allows adequate placement of the probe for electroejaculation [34,35].

**Figure 5:**
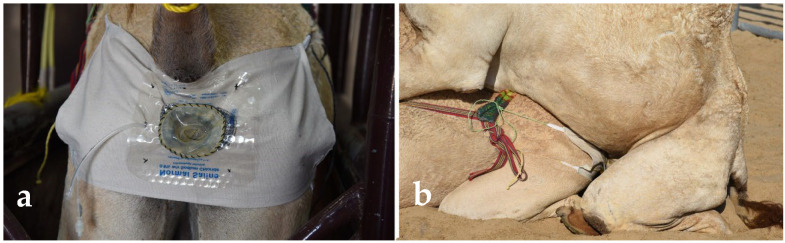
Semen collection in dromedary using specially designed latex vaginal condom. (**a**) female prepared for semen collection by placing a vaginal condom; (**b**) mating with a female having vaginal condom.

Semen evaluation, an essential component of the BSE, is difficult in the field due to the challenges in reliably obtaining a representative semen sample. The use of an artificial vagina requires long periods of male training and is not always as successful as it has been reported in the literature [5]. Electroejaculation has been described for all camelid species [3,16]. The procedure requires general anesthesia, presents welfare issues, and is not acceptable to camel owners. In SACs, the method used in the field by most veterinarians is postcoital semen aspiration [36]. This technique provides information on sperm motility and morphology, but is of no value for the determination of sperm concentration and total sperm output. Additionally, semen samples are often contaminated with blood from the females. In camels, a recently described semen collection technique using a latex vaginal condom (Figure 5) offers a more practical approach and is readily accepted by males [5].

Another challenge in camelid semen evaluation is its viscous nature, which hinders the use of standard techniques to evaluate sperm motility, morphology, and concentration. Liquefaction of the ejaculate must be completed before these tests can be conducted accurately. Liquefaction cannot be obtained entirely by simple incubation of semen as proposed by some authors. Liquefication is obtained by enzymatic (papain or collagenase) [37,38,39] or physical (pipetting [5], cushion or single layer centrifugation [40,41] treatment. Even with these procedures, a complete liquefaction may not be achieved in some males. Without complete liquefaction, the determination of progressive motility, sperm motion parameters, and sperm concentration is not accurate. These issues with semen liquefaction are probably one of the significant factors in the variability observed in the published camelid ejaculate characteristics. Another challenge in semen evaluation is the presence of froth in collected ejaculates [5,42].

The breeding soundness examination is mostly a method to distinguish between animals that are potential satisfactory breeders from those that are unsatisfactory. To determine the cause of infertility, other examination techniques such as endocrinology, histopathology, and cytogenetic evaluation are necessary [16,43,44,45]. Abnormalities of the reproductive system found by the authors on routine breeding soundness examination or examination of infertile males are presented in Table 1. A total of 48.84% of the alpacas submitted for regular breeding soundness examination had an abnormality of the reproductive system reinforcing the importance of prebreeding or prepurchase examination. As expected, the incidence of genital tract abnormalities was higher in males presented for infertility (90.32% of alpacas, 83.33% of camels). However, it is important to note that 10% of alpacas and 17% of camels presented for infertility did not show any abnormalities on BSE. This suggests that some cause of infertility may not be evident on clinical examination only and may require more advanced evaluation techniques (biopsy, cytogenetics, biochemical analysis). It is important to note that a large proportion of males presented for infertility do not display any obvious changes on examination of the genitalia.

Reproductive disorders described in camelids can be grouped in 4 broad categories: (1) congenital defects of the reproductive system, (2) *impotentia coeundi* (inability to copulate), (3*) impotentia generandi* (inability to impregnate fertile females after normal mating), and (4) reproductive emergencies.

## 3. Congenital Abnormalities

### 3.1. Cryptorchidism

For SACs, there is a unanimous agreement that the testes should be in the scrotal position at birth. Testicular migration into the scrotal position is not well studied in camels. The age at which the testes should be in the scrotal position varies from 1 to 4 years, depending on studies. Preliminary data from the Advanced Scientific Group, Veterinary Research Center, UAE show that all yearlings or older should have scrotal testis (Anouassi A, unpublished). This observation, as well as the ability to castrate dromedary calves as early as 6 months (Tibary A, clinical observation), suggests that testicular descent into the scrotum in camels occurs a lot earlier than previously reported.

**Figure 6:**
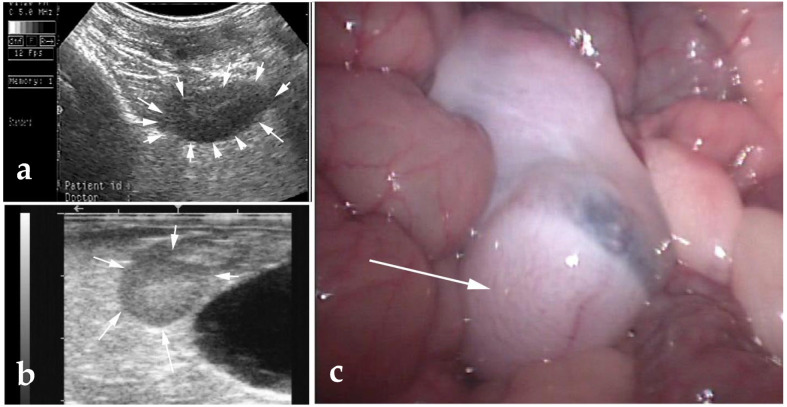
Confirmation of cryptorchidism by transabdominal ultrasonography (**a**), transrectal ultrasonography (**b**), and laparoscopy (**c**). Arrow(s) point to a retained testis.

Cryptorchidism has been described in all camelids, including Bactrian camels [46,47,48] and vicuñas [49,50]. Abattoir studies reported an incidence of 3% in alpacas [14] and 0.7–1.3% in dromedaries [13,51]. As described by other authors, all the retained testes in our cases were abdominal, often close to the vaginal ring, and sometimes caudal to the kidneys [47,48,50,52]. Confirmation of the diagnosis and location of the retained testis are achieved by transabdominal or transrectal ultrasonography and laparoscopy [50,52,53] (Figure 6). Intraabdominal testes may be associated with XX sex-reversal syndrome [54]. In rare cases in alpacas, one of the testicles may be completely absent (monorchidism), and the disorder has been associated with ipsilateral kidney agenesis [14].

In animals that are presumably castrated but demonstrate male behavior, the presence of testicular tissue is confirmed by a rise in serum testosterone levels in response to administration of human chorionic gonadotropin (hCG) (3000 IU in llamas and alpacas, 6000 IU in camels, IV). A two-fold increase in serum testosterone concentration 8 h following administration of hCG confirms the presence of testicular tissue [50,55]. Serum concentration of Anti-Müllerian hormone, a hormone produced only by Sertoli cells in the male, is a reliable indicator of the presence of testicular tissue [50,56].

Cryptorchid males should be castrated because the disorder is possibly hereditary [49,57], and retained testicles are predisposed to neoplasia [58].

### 3.2. Ectopic Testis

**Figure 7:**
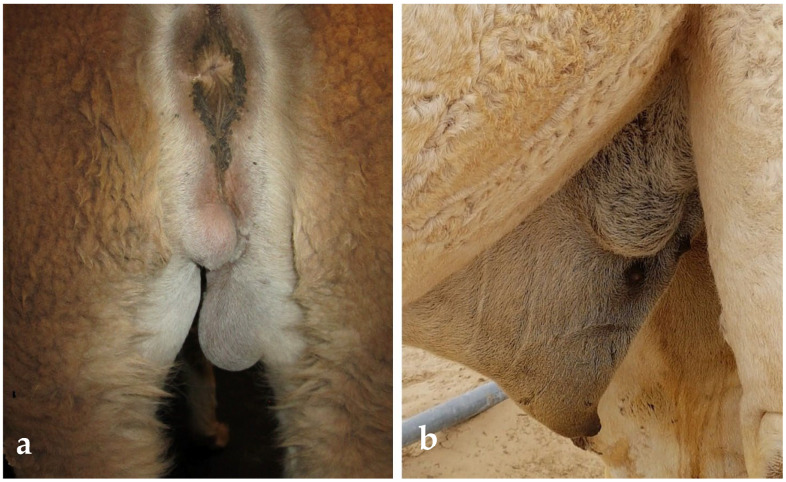
An ectopic testis in a llama (**a**) and in a dromedary (**b**).

The abnormal location of the testis outside the abdominal cavity, testicular ectopia, is more common in SACs than in camels [14]. The testicle is often found subcutaneously along the prepuce or the medial aspect of the leg [14,36] (Figure 7). Males with ectopic testicles may be presented for examination because of abnormal gait or pain during mating. Unilaterally affected animals are still fertile but should be eliminated from reproduction.

### 3.3. Testicular and Epididymal Cysts

**Figure 8:**
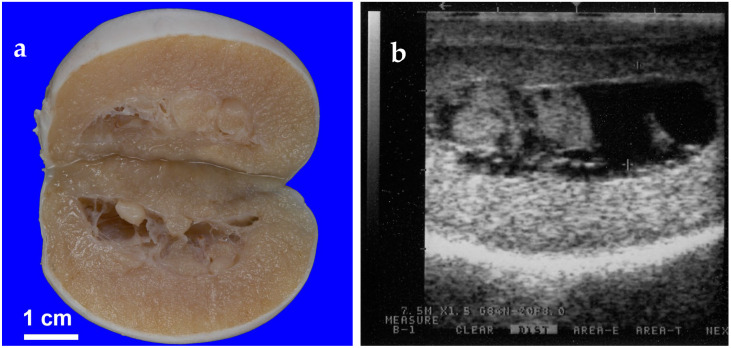
Gross (**a**) and ultrasonographic (**b**) appearance of rete testis cyst in an alpaca.

Bilateral testicular and epididymal cysts have been reported as a cause of infertility in alpacas and llamas [59,60]. Congenital cystic dilation of the rete testis (rete testis ectasia) is the most common (Figure 8). An incidence of 14.5% has been reported in an abattoir study of alpacas [14]. A high incidence has also been reported in alpacas presented for breeding soundness evaluations or infertility (Table 1). Rete testis ectasia is less common in camels [61]. On ultrasonography, these cysts range in size from a few millimeters to several centimeters and may extend to the head of the epididymis. In our cases, 44.4% of fluid aspirated rete testis cysts contained immature spermatozoa. Bilaterally affected males with large cysts are oligo- or azoospermic [62]. Congenital rete testis cysts are due to abnormal development of the efferent ductules or tubuli recti or the terminal end of the seminiferous tubules or ectopic mesonephric epithelium. Congenital segmental aplasia and cystic dilation of the epididymis have been described in SACs, and is associated with infertility [14,45,60,63].

### 3.4. Testicular Hypoplasia

**Figure 9:**
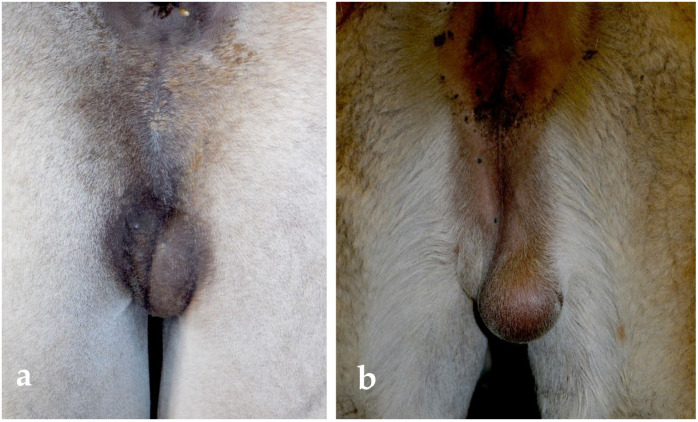
Unilateral testicular hypoplasia in a dromedary (**a**) and in an alpaca (**b**).

The reported incidence of testicular hypoplasia is 8 to 10% in SACs [14,45] and 1.6% in camels [12,51,64]. It can be unilateral, resulting in asymmetric development of the testicles, or bilateral with various degrees of severity (Figure 9). Ejaculates of the affected males can be oligozoospermic, teratozoospermic, or azoospermic. Testicular size is often more than 20% below the average for the age. Libido is generally not affected [61].

**Figure 10:**
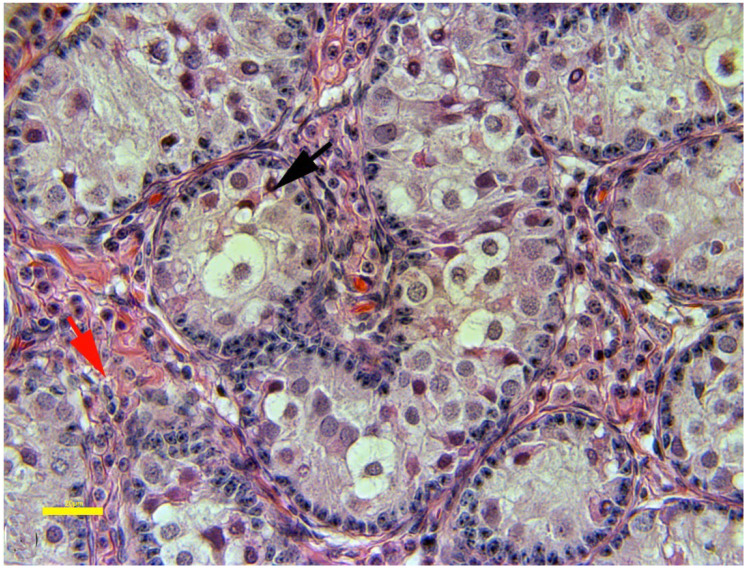
Histologic view of severe testicular hypoplasia in an alpaca showing normal Leydig cells (red arrows) and seminiferous tubules devoid of spermatogenesis having only Sertoli cells (black arrow).

In SACs, testicular hypoplasia is suspected to be hereditary and may be associated with cytogenetic abnormalities. Differential diagnosis between congenital testicular hypoplasia and severe testicular degeneration requires evaluation of testicular fine needle aspirates or biopsy (Figure 10). Histological examination of the hypoplastic testes reveals a lack of or reduced spermatogenesis. In some cases, seminiferous tubules may be devoid of spermatogonia (Sertoli cell only syndrome) [65].

### 3.5. Preputial and Penile Developmental Abnormalities

Abnormalities of the development of the prepuce and penis have been described in males with abnormal external appearance or dysuria [66]. Several cases of sexual development disorders are XX sex-reversed animals. They are identified as females at birth and then start displaying male-like behavior around puberty because of having bilateral abdominal testes [66].

Phimosis or a persistent penile frenulum prevents normal extension of the penis during erection. Surgical correction is possible, but affected males should be removed from breeding because the traits are suspected to be hereditary. Other rare abnormal developments of the prepuce and penis include a short penis (micropenis), hypospadias [67], and incomplete development of the glans penis [21,67].

## 4. Causes of *Impotentia Couendi*

*Impotentia coeundi* is defined broadly as the inability of the male to complete mating. It can be due to poor libido, failure to achieve a normal erection, or complete intromission.

Poor libido is a common complaint in the male camelid. Male camels, being seasonal breeders, have reduced serum testosterone levels during the non-breeding season. In the camel racing industry, breeding is often initiated before the natural breeding season when the libido is physiologically low but may be perceived as abnormal. Testosterone production in subordinate males may be reduced when housed in proximity to dominant males. Although administration of GnRH can enhance libido and even semen quality [68], it is essential to consider other causes that may reduce libido, such as overbreeding and systemic diseases. In the racing industry, many males are mated up to 10 times per day and cannot sustain this breeding frequency. The appropriate frequency of mating per day has not been established scientifically in the dromedary. In South American camelids, mature males can sustain 4 matings per day for a period of 10 days [69]. Reduced libido is also one of the first clinical signs of severe diseases, such as trypanosomiasis. Some behavioral disorders may interfere with reproductive behavior and libido. Camels can develop stereotypical behavior such as pacing, continuous rubbing of the poll glands against fences or walls, ground-breeding, and self-mutilation (biting of the legs and flank) [70]. This is often observed in males that are housed near each other. Excessive rubbing against fences may lead to severe inflammation of the poll glands [61].

**Figure 11:**
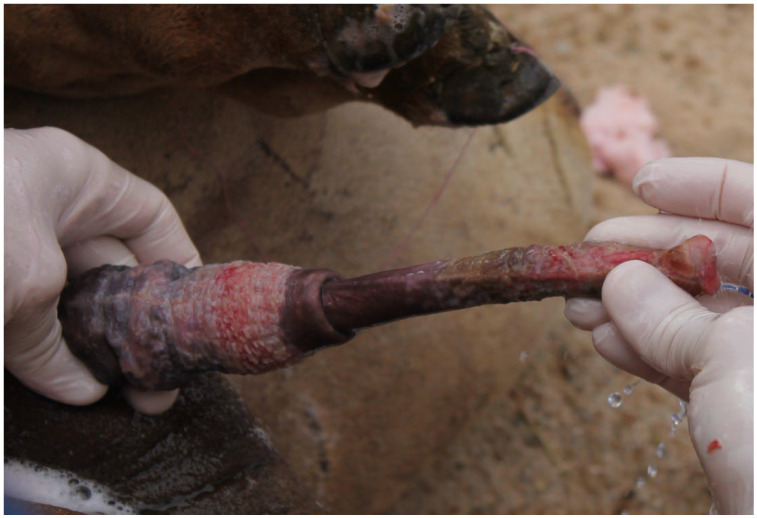
Ulcerative balanoposthitis in a dromedary camel caused by placement of a tight preputial ring (to prevent masturbation) and urine accumulation.

Other causes of impotentia coeundi include painful conditions such as balanitis or balanoposthitis (Figure 11). These conditions can also be associated with abnormal behavior during mating, such as savaging the female or self-mutilation. Ulcerative balanoposthitis commonly results from masturbation. Most cases of phimosis in breeding males are associated with ulcerative posthitis, penile lacerations, and adhesion formation [50,61,65].

## 5. *Impotentia Generandi*

*Impotentia generandi* is defined as the inability of a male to achieve pregnancy in fertile females despite successful mating. In general, the low pregnancy rate observed is due to ejaculation failure (aspermia) or poor quality of the ejaculate (oligo- or azoospermia, teratozoospermia, asthenozoospermia).

The ejaculatory process in camelids is continuous as the semen is emitted in successive small quantities throughout the mating period [71,72]. It is challenging to ascertain ejaculation failure (aspermia, absence of ejaculate) in camelids owing to the method used for semen collection. Lack of ejaculation is often observed as a poor response to electroejaculation and the artificial vagina (mounting without ejaculation) [5]. Incomplete ejaculation can also result from interruption of copulation due to painful conditions.

**Figure 12:**
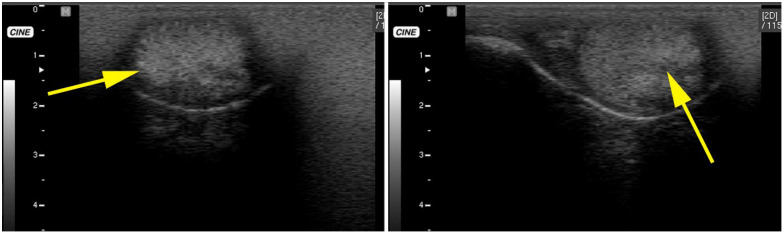
Ultrasonographic appearance of bilateral testicular degeneration in a 7 years old alpaca. Arrows indicate hyperechogenic fibrotic mineralized areas. Note the small size of the testes for an adult alpaca.

Oligozoospermia, azoospermia, and teratozoospermia are often observed in cases of infertility/subfertility in the male. The leading cause of reduced quality of the ejaculate is testicular degeneration [45,61,73,74]. The clinical hallmarks for testicular degeneration are testicular asymmetry if unilateral and overall reduced testicular size if bilateral degeneration occurs. On palpation, the testes may be soft or hard. Ultrasonography may reveal areas of fibrosis or mineralization in severely atrophied testes (Figure 12).

**Figure 13:**
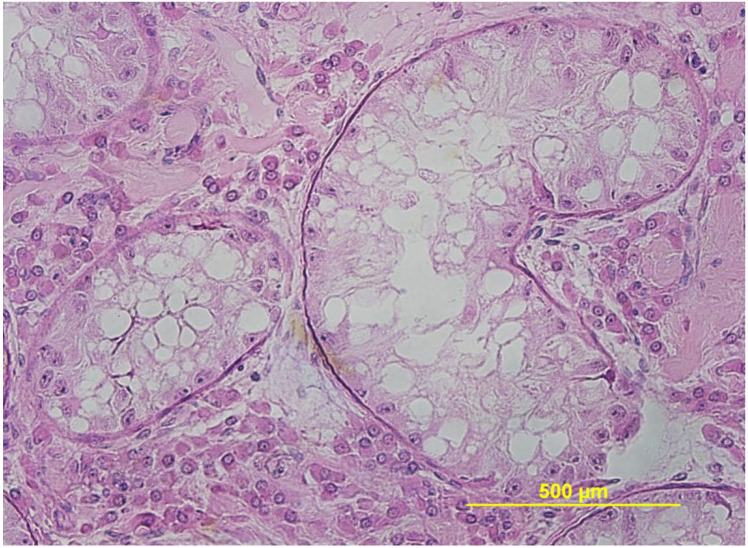
Histological appearance of severe testicular degeneration in a llama. The seminiferous tubules are larger in diameter, lack spermatogenesis and are vacuolated.

Testicular biopsy is the gold standard for confirmation of the diagnosis and reveals low or absent spermatogenic activity, collapsed vacuolated seminiferous tubules (Figure 13), and/or the presence of fibrous tissue and inflammatory cell infiltration [17]. Camels with impaired spermatogenesis, oligozoospermia or azoospermia, have high serum FSH, LH and testosterone levels. In camelids, unlike stallions and dogs, the determination of seminal plasma alkaline phosphatase concentration is not a good method to differentiate between primary (lack of spermatogenesis) and secondary (failure of sperm transport) azoospermia, because the enzyme is produced by the accessory sex glands [34].

In dromedary camels, testicular degeneration was reported in 33.5% of examined testes in an abattoir study [13]. The incidence of testicular degeneration increases with age (>15 years in camels, >10 years in SACs) and following testicular insults (hemorrhage, orchitis) [13,45,63]. In the dromedary camel, testicular degeneration, oligozoospermia, and teratozoospermia are observed following chronic administration of testosterone to increase libido [64]. Testicular degeneration is also seen in racing and show dromedaries treated with anabolic steroids. Trypanosomiasis was shown to induce the formation of an immune complex precipitate under the basement membrane of the seminiferous tubules, disrupting spermatogenesis and Sertoli cell function. These changes result in endocrine dysfunction, ultimately leading to severe testicular degeneration [64,75]. In SACs, in addition to traumatic and infectious insults, heat stress is an important cause of testicular degeneration [45].

Other causes, such as trace mineral deficiencies (selenium and zinc), have been suspected in cases of testicular degeneration. Administration of zinc, selenium and vitamin E improved semen quality of dromedary males with *impotentia generandi* [76].

## 6. Male Reproductive Emergencies

The most common reproductive emergencies in the male camelids are scrotal enlargement, penile or preputial trauma, preputial prolapse, and urolithiasis. In breeding camels, two other emergencies associated with reproductive activity are soft palate (dulla) trauma and rectal prolapse [50,77].

### 6.1. Scrotal Enlargement

Scrotal enlargement may be due to trauma or inflammation and may be acute or progressive. Although not sudden, scrotal enlargement is observed with testicular neoplasia. In SAC, heat stress is a common cause of scrotal enlargement due to a hydrocele [61].

**Figure 14:**
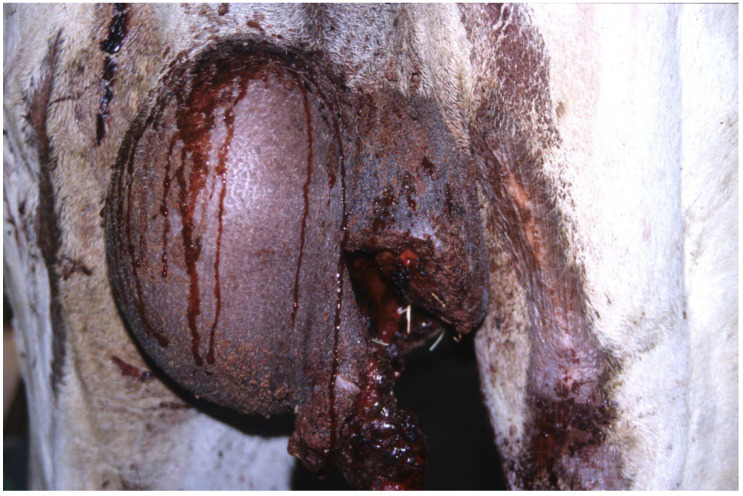
Scrotal trauma in a dromedary.

Scrotal trauma and testicular hemorrhage are common consequences of fighting among males [61] (Figure 14). These injuries are extremely painful and require emergency (unilateral if possible) orchiectomy. It is important to note that testicular hemorrhage may be observed in some cases of testicular neoplasia and orchitis.

**Figure 15:**
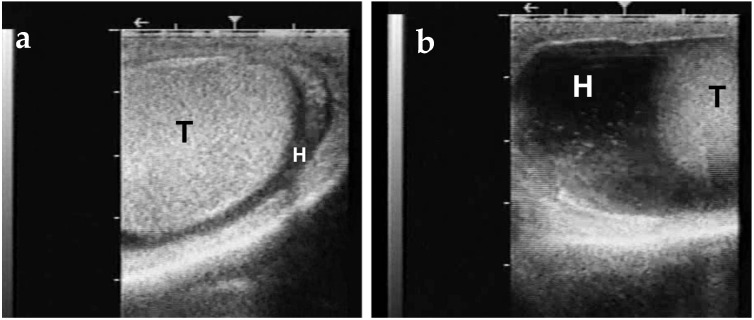
Both (**a**) and (**b**) demonstrate ultrasonographic appearance of hydrocele in dromedary camel. T represents testis, H represents fluid accumulated in the hydrocele.

Inflammatory processes leading to scrotal enlargement include dermatitis, orchitis, and epididymo-orchitis. Scrotal dermatitis is often observed with mange or tick infestation. These lesions are usually accompanied by hydrocele (Figure 15), hematocele, or pyocele [61].

In dromedary camels, abattoir studies reported an incidence of orchitis ranging from 2.3% to 10% [13]. Bacterial isolates from orchitis include *Corynebacterium pseudotuberculosis*, *Pseudomonas* [13] *aeruginosa*, *Chlamydophila (Chlamydia) abortus*, *Mycoplasma* spp. *Mycobacterium tuberculosis*, *Brucella abortus* and *Brucella melitensis* [78,79,80]. Parasitic (filariasis due to *Dipetalonema evansi*) orchitis was found in 7.7% of examined testicles from slaughtered camels in Egypt and Iran [81,82]. *D. evansi* filariae are found in the arteries of the spermatic cord, testes, and epididymides, inducing vasculitis and perivasculitis [83]. Orchitis has been observed as a complication of scrotal sarcoptic mange in camels [79]. The role of viruses in camelid orchitis is not clear. A possible involvement of the blue tongue virus and the camel pox virus is suspected but needs further investigation.

**Figure 16:**
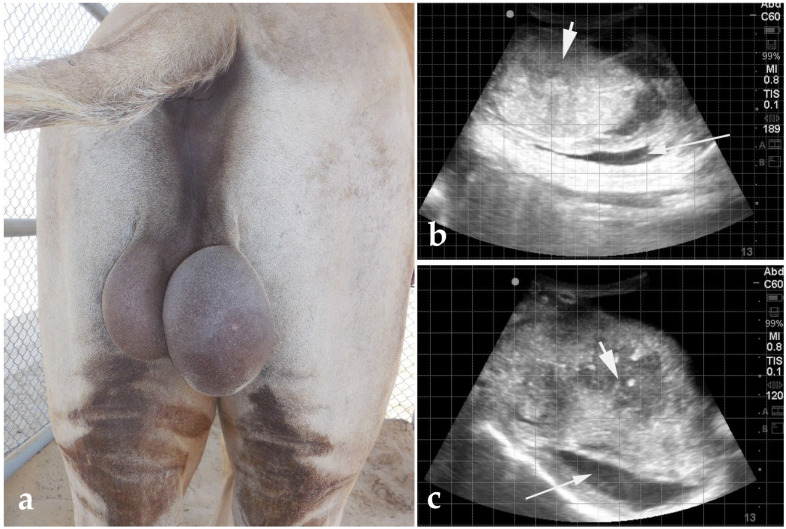
Gross (**a**) and ultrasonographic (**b**,**c**) appearance of orchitis caused by Brucellosis in a dromedary camel. Ultrasonography shows that the scrotum is thickened and the parenchyma has an abscess (thick arrowhead). Thin arrows point to concurrent hydro/pyocele.

Orchitis is probably underestimated in SACs. Most of the cases seen by the authors are discovered on histological evaluation of atrophied testis. These were suspected to originate from a local injury or septicemia, primarily due to *Streptococcus equi zooepidemicus* [84]. The camelids are susceptible to brucellosis due to *B. abortus* and *B. melitensis*, which can be a cause of epididymo-orchitis (Figure 16) in countries where the disease is endemic. Diagnosis of orchitis is based on clinical signs, palpation, and ultrasonography. In acute orchitis, the scrotum is swollen, warm, and painful. Scrotal ultrasonography often reveals various quantities of hydrocele or pyocele, and heterogeneous testicular parenchyma with sometimes abscessation (Figure 16). The scrotal skin and vaginal tunic are thick and occasionally present fibrinous material, suggesting a periorchitis. A nodular appearance, atrophy, increased hardness, presence of adhesions, and testicular mineralization characterize chronic orchitis [61].

Treatment of orchitis is often unrewarding. Castration is the best course of action and may salvage the breeding value of the animal if the condition is unilateral and not due to a contagious disease. Camels with orchitis should be tested for brucellosis and eliminated from the herd if positive.

Non-inflammatory hydrocele is a common finding in hot and humid environments, particularly in SACs. The condition resolves progressively with decreasing ambient temperature, but the quality of semen may remain suboptimal (high incidence of abnormalities and low motility) for up to 3 months. Hydrocele represents an important physical finding in depressed llamas and alpacas, as it is a primary clinical sign of heat stress. In these cases, the change in scrotal size is often discovered at the time of shearing [77].

Scrotal enlargement due to inguinal/scrotal hernia is rare in camelids. Bilateral indirect inguinal hernia was reported in a six-year-old alpaca with severe weight loss due to heavy *Haemonchus contortus* infestation [85]. Hernia content (intestines) can be palpated or visualized by ultrasonography.

**Figure 17:**
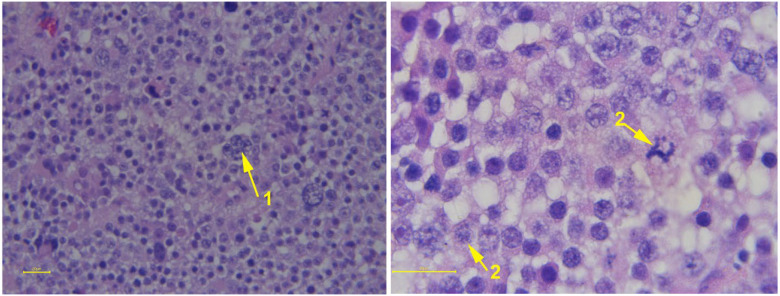
Histological appearance of seminoma from an alpaca. The majority of the cells are densely packed, polygonal-shaped mononuclear cells. Some multinucleated cells (1) are also seen besides mitotic figures.

Scrotal enlargement due to testicular tumors is usually gradual. Testicular neoplasia reported in camelids includes seminomas [45,86,87], Sertoli cell tumors [88,89], and teratomas [63]. Testicular tumors are generally unilateral. Their effect on behavior and fertility depends on their endocrine activity. A shift in the offspring sex ratio in favor of females (97%) was reported in a dromedary with unilateral seminoma [87]. Ultrasonography may reveal a lobulated appearance and loss of normal contour of the mediastinum testis. Hemicastration is recommended in unilateral cases. Diagnosis is confirmed by fine needle aspirate or histopathology after castration (Figure 17). Metastasis to other organs has not been reported.

### 6.2. Preputial/Penile Injuries

Preputial swelling may be due to local inflammation caused by contact with chemicals or physical irritants, parasitic infestations (tick, mange), or the introduction of debris into the prepuce during masturbation. In camels, preputial swelling may develop following overuse in breeding. Preputial swelling may also be part of extensive ventral edema in some animals suffering from heat stress (SACs) or acute trypanosomiasis in camels [77,90].

**Figure 18:**
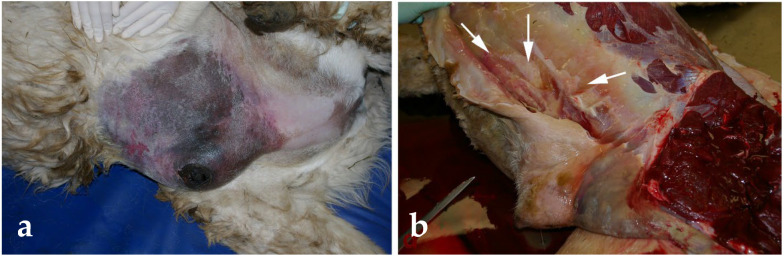
(**a**) Preputial edema, severe cellulitis and necrosis from infiltration of urine from ruptured urethra in an alpaca. (**b**) Postmortem examination showing infiltration of subcutaneous tissue with urine (white arrows).

Any acute preputial swelling should be considered an emergency [77,90]. In addition to a complete physical examination, baseline complete blood count and blood biochemistry, the initial examination should include evaluation of urination and ultrasonographic assessment of the swelling, as well as transrectal imaging of the bladder. Prognosis is guarded to poor if there is evidence of a ruptured urethra. Subcutaneous infiltration of urine from a ruptured urethra often results in severe cellulitis and eventually loss of tissue vitality (Figure 18). A thorough evaluation of the penis should be performed in case of preputial swelling or the presence of abnormal preputial discharge [61]. The penis can be exteriorized under general anesthesia or following pudendal nerve block in camelids [33]. Abscesses and adhesions formation are common following preputial and penile laceration, often leading to paraphimosis. In camels, preputial injuries are usually associated with tight girth straps in riding or working animals, which can cause paraphimosis and tissue necrosis. Medical management of these cases is often unrewarding, and phallectomy is the only option to salvage the male, but the reproductive function is lost.

### 6.3. Preputial Prolapse

**Figure 19:**
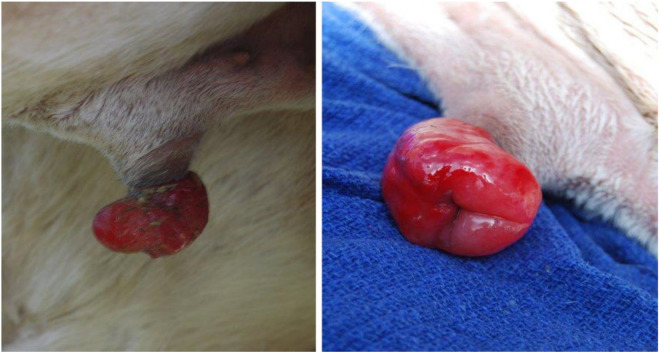
Gross appearance of an edematous preputial prolapse in an alpaca due to ground breeding.

Preputial prolapse (Figure 19) is often a complication of edema or repeated irritation caused by frequent masturbation (breeding sand or objects).

**Figure 20:**
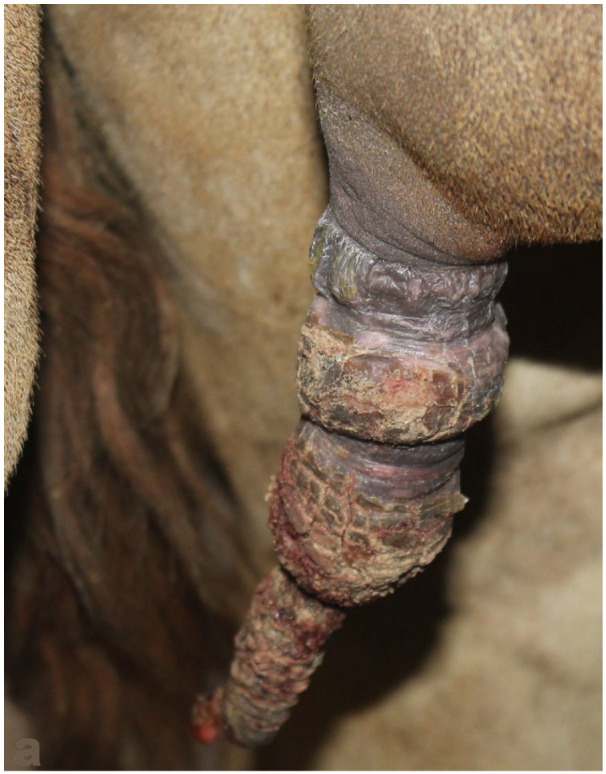
Paraphimosis in a dromedary camel showing excoriation and necrosis of tissue.

**Figure 21:**
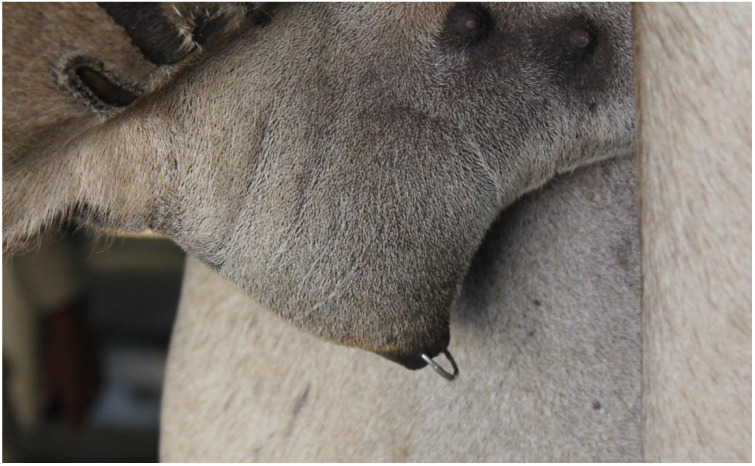
Placement of preputial ring in a male dromedary to prevent masturbation.

Early cases may be corrected by replacing the prolapsed tissue and placing purse-string sutures for a few days [50]. The preputial edema can be reduced by bandaging using a stockinette after application of an emollient (500 g anhydrous lanolin, 60 mL scarlet oil, 2 g tetracycline powder) [2]. Complications of untreated preputial prolapse include paraphimosis, severe adhesions, and compromised urine flow leading to tissue necrosis (Figure 20). These cases have a guarded to poor prognosis for return to normal reproductive activity. Preputial rings (Figure 21) are commonly used to prevent masturbation in dromedaries, but they can lead to complications.

### 6.4. Urolithiasis

Urolithiasis is a common problem in the male camelid with stranguria and abdominal discomfort [61,91,92]. Physical examination may reveal tachypnea, tachycardia, dark mucous membrane, and increased capillary refill time. On ultrasonography, the bladder and sometimes the ureters are distended [93]. Uroliths may be visualized by ultrasonography or radiography (SACs). Various approaches for the medical and surgical management of these cases have been described [94]. However, in most cases, the reproductive ability of the male is lost. If not treated promptly, severe electrolyte imbalances and uremia lead to a deterioration of the animal’s health.

Prevention of urolithiasis is based on proper nutritional management, through feeding a balanced ration, salt supplementation to acidify urine, prevention of precipitation, and good access to fresh water. Supplementation with ammonium chloride has been used in alpacas and llamas (5–10 g/kg) to reduce struvite crystals [95]. In the camel, urolithiasis is more frequently observed in animals fed a diet rich in grain and rarely observed in animals on desert pastures, presumably because of access to natural plants with high salt content.

### 6.5. Prolapse of the Soft Palate in the Dromedary

**Figure 22:**
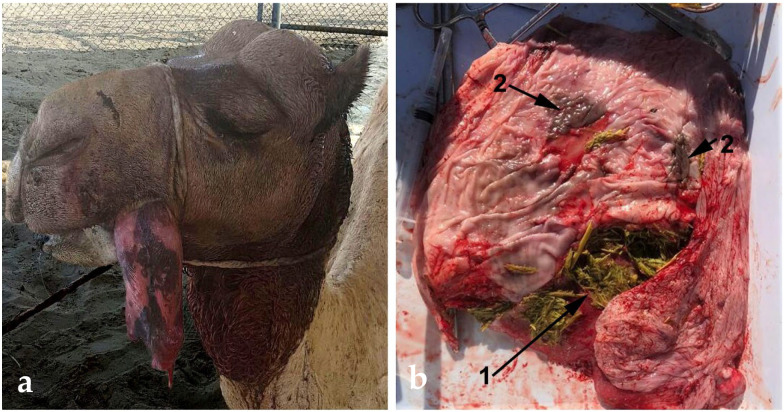
(**a**) Exteriorized dulla in a male dromedary; (**b**) Resected dulla from a rutting dromedary camel having impacted food (1) and traumatic lesions (2).

Impaction and trauma of the soft palate (dulla), as well as trauma and inflammation, are common problems in the dromedary during the rutting season [50,96,97,98,99]. The traumatic lesions are rapidly complicated by severe inflammation and infection. The inflamed organ becomes permanently exteriorized on the side of the mouth (Figure 22), causing further tissue damage and necrosis [35]. In untreated cases, the health of the animal deteriorates because of anorexia and impairment of mastication and deglutition. Sudden death due to asphyxiation has also been reported [100]. Palatectomy is the best treatment [35,96,99,101]. This condition is not seen in breeding racing males because palatectomy is often performed early during training to improve performance.

## 7. Rectal Prolapse

**Figure 23:**
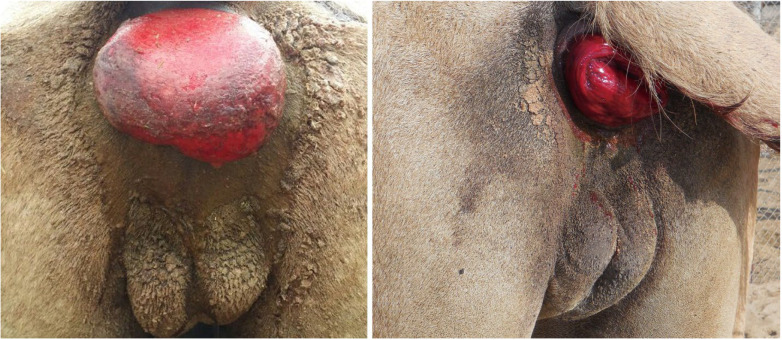
Rectal prolapse in male dromedary camels.

Rectal prolapse associated with breeding activity is primarily described in camels [50,102] (Figure 23), but has also been reported in a llama [103]. Conservative management (replacement and purse string suture) is successful when the prolapse is intermittent, but surgical repair (mucosal resection and anastomosis or rectal amputation) is necessary in chronic cases [50,96,104].

## 8. Other Causes of Male Infertility

A high proportion of male infertility remains undiagnosed. A substandard biological, cytogenetic, and microbiological semen quality may be involved in these cases.

The healthy camelid prepuce harbors diverse microorganisms. Disruption of the balance of this flora may lead to transmission of pathogenic bacteria to females [105,106] . Ureaplasma and *Mycoplasma* organisms have been isolated in 30 to 40% of preputial samples in camels [107]. In a study on 141 infertile dromedary males, semen parameters were negatively correlated with positive titers for *Chlamydia abortus* or culture of *C. fetus* [108]. *Campylobacter* spp. and *Tritrichomonas foetus* have been isolated from preputial scrapings. Campylobacteriosis and trichomoniasis are known to be asymptomatic in camel bulls but have been incriminated in infertility in dromedary females in camels [109]. Campylobacter spp. have been isolated from aborted alpacas; however, the venereal transmission has not been confirmed [110].

In one study of infertile camels, 88% of semen samples were positive by PCR for *Mycoplasma* spp., *Leptospira* spp., *Brucella melitensis*, or Bovine Viral Diarrhea (BVD) virus, suggesting that these organisms may be involved in male infertility [111]. In alpacas, the BVD virus was demonstrated in various tissues, including the reproductive organs of experimentally infected and persistently infected animals [112,113]. The role that the male may play in the venereal transmission of these organisms, as well as others known to cause early embryonic death or abortion, needs further evaluation.

Spermatogenesis and semen quality are affected by several trace minerals. Selenium and zinc deficiency were shown to reduce libido and impair spermatogenesis and sperm quality, most likely through an increase in oxidative damage [114]. Administration of vitamin E (α-tocopherol acetate, 1 mg/kg bw, IM), selenium (sodium selenite, 0.088 ng/kg bw, IM) weekly for 3 weeks and daily oral supplementation of zinc fluconate (360 mg) for 5 weeks improved semen quality parameters and conception rate of infertile dromedary camels [76]. Serum Cadmium levels were negatively correlated with sperm concentration, testicular size, motility, and viability, but positively correlated with sperm abnormalities [115]. High concentrations of heavy metals (arsenic, cadmium, and lead) were found in the seminal plasma and serum of infertile dromedary males compared to fertile males. They were negatively correlated with sperm motility and concentration [116]. These are interesting observations, but a more thorough evaluation of trace mineral supplementation on sperm quality is needed.

Fertility biomarkers may shed some light on unexplained infertility in males. Studies have shown a positive correlation between dromedary camel fertility and seminal plasma concentration of osteopontin (a matricellular protein that mediates various biological functions) [117]. On the other hand, serum malonialdehyde (biomarker of oxidative stress), insulin-like growth factor1, prostaglandin D synthase, and clusterin (apolipoprotein associated with apoptosis) are all negatively correlated with fertility [117].

The expression of several genes that regulate sperm function (PLA2G4D, SPP1, and CLUAP1) was significantly higher in the testes of dromedary males with high cauda epididymis sperm quality. A significant difference in the expression of several genes involved in material transport and the testicular defense mechanism was also reported [118,119]. ProAKAP4 (A-kinase anchor protein 4), a functional marker of spermatozoa, is localized in the principal piece of the flagellum of the dromedary sperm. The concentration of ProAKAP4 was found to be positively correlated with ejaculate volume, viscosity, and sperm motility, and could provide more insight into fertility [120].

In our embryo transfer program, one male with all semen parameters within normal range failed to produce any embryos following natural cover. This suggests that other factors (immunological or genetic) may be involved in male infertility in camelids. In the dromedary, we have suspected that breeding frequency may affect sperm concentration and/or levels of ß-Nerve Growth Factor (ß-NGF) in the ejaculate, resulting in a decrease in the conception rate. However, a recent study on alpacas did not find any correlation between ß-Nerve Growth Factor concentration in semen and fertility [42].

There is a lack of studies on the role of cytogenetic problems in male infertility, especially in camels. An autosomal abnormality (translocation between chr 12 and chr 20) has been reported in a sterile llama with teratozoospermia [121]. The role of cytogenetic abnormalities in the failure of fertilization or early embryonic death merits more investigation [43].

## 9. Conclusions

Natural mating remains the most common method of reproduction in camelids, making male fertility an essential factor in herd reproductive efficiency. Significant advances have been made in the study of male camelid reproduction. Examination techniques established in other species for the investigation of reproductive function have been applied to camelids. Clinical techniques such as testicular ultrasonography, fine needle aspirate, and biopsy are well established. However, the basic information that relates findings of breeding soundness examination to fertility is still scarce, especially in camels. This is in part due to the variety of types or breeds of camels and herd management systems.

Additionally, regular male breeding soundness evaluations are not emphasized in veterinary camelid practice. The development of simple methods for semen collection in the field will be beneficial for the incorporation of male BSE into the practice. The study of other aspects of male fertility is either incomplete (i.e., endocrinology) or lacking (i.e., cytogenetics).

It is important to note that male fertility may be affected by disease prevalence and several management factors, including nutrition, proper housing, and female/male ratio. Veterinarians need to be trained on appropriate methods to examine all these factors when investigating poor reproductive performance in a herd. Finally, camelid owners should be alerted to the importance of preventing male reproductive disorders through proper health care, nutrition, and housing.

## Figures and Tables

**Table 1:** Incidence of abnormalities of the reproductive systems in alpacas and dromedary camelids submitted for breeding soundness examination or infertility (excluding emergencies).

Condition	Alpacas		Dromedary Camels
	BSE (N = 301)	Infertility (N = 93)	Infertility (N = 48)
	N (%)	N (%)	N (%)
Peno-preputial attachment/frenulum	9 (2.99)	-	1 (2.08)
Phimosis/adhesions	-	4 (4.30)	3 (0.63)
Cryptorchidism	4 (1.33)	-	-
Ectopic testicle	3 (0.99)	1 (1.07)	1 (2.08)
Rete testis cysts (unilateral)	30 (9.97)	9 (9.68)	-
Rete testis cysts (bilateral)	48 (15.95)	16 (17.20)	1 (2.08)
Epididymal cysts	1 (0.33)	4 (4.30)	1 (2.08)
Testicular hypoplasia	24 (7.97)		2 (4.16)
Testicular degeneration/atrophy	15 (4.98)	33 (35.48)	21 (43.75)
Orchitis	3 (0.99)	8 (8.60)	7 (14.58) *
Hydrocele	10 (3.32)	9 (9.67)	3 (6.25%)
Total abnormalities	147 (48.84)	84 (90.32)	(83.33)

* All 7 were positive for brucellosis.

## Data Availability

Data is contained within the article however any additional dataset/information available on request from the authors.
